# High capacity for a dietary specialist consumer population to cope with increasing cyanobacterial blooms

**DOI:** 10.1038/s41598-022-26611-2

**Published:** 2022-12-22

**Authors:** Matias Ledesma, Elena Gorokhova, Andrius Garbaras, Linda Röjning, Beatriz Brena, Agnes M. L. Karlson

**Affiliations:** 1 Department of Ecology, Environment and Plant Science (DEEP), Stockholm University, Svante Arrhenius St. 20, Stockholm, Sweden; 2 Department of Environmental Science, Stockholm University, Svante Arrhenius St. 8, Stockholm, Sweden; 3 Department of Nuclear Research, Centre for Physical Science and Technology, Savanorių Ave. 231, Vilnius, Lithuania; 4 Department of Biosciences, University of the Republic, Gral. Flores 2124, 11800 Montevideo, Uruguay; 5 Baltic Sea Centre, Stockholm University, Svante Arrhenius St. 20, Stockholm, Sweden; 6 Bolin Centre for Climate Research, Stockholm University, Svante Arrhenius St. 8, Stockholm, Sweden

**Keywords:** Boreal ecology, Climate-change ecology, Ecophysiology, Stable isotope analysis

## Abstract

We present a common-garden experiment to examine the amphipod *Monoporeia affinis*, a key deposit-feeder in the Baltic Sea, a low diversity system offering a good model for studying local adaptations. In the northern part of this system, the seasonal development of phytoplankton is characterized by a single diatom bloom (high nutritional quality), whereas in the south, the diatom bloom is followed by a cyanobacteria bloom (low nutritional quality) during summer. Therefore, the nutrient input to the benthic system differs between the sea basins. Accordingly, the amphipod populations were expected to be dietary specialists in the north and generalists in the south. We tested this hypothesis using a combination of stable isotope tracers, trophic niche analyses, and various endpoints of growth and health status. We found that when mixed with diatomes, the toxin-producing cyanobacteria, were efficiently incorporated and used for growth by both populations. However, contrary to expectations, the feeding plasticity was more pronounced in the northern population, indicating genetically-based divergence and suggesting that these animals can develop ecological adaptations to the climate-induced northward cyanobacteria expansion in this system. These findings improve our understanding regarding possible adaptations of the deposit-feeders to increasing cyanobacteria under global warming world in both limnic and marine ecosystems. It is possible that the observed effects apply to other consumers facing altered food quality due to environmental changes.

## Introduction

Predictions about the consequences of a rapidly changing climate for populations need to consider the potential for affected organisms to acclimate to the stressor during the lifespan of an individual, allowing them to maintain growth via adjusting metabolic processes. In addition, it is important to realize that locally adapted organisms across persistent environmental gradients may vary in their response to stressors (e.g., novel resources, toxicity).

Cyanobacterial blooms are increasing globally in a warmer climate, and since some species are toxic, the blooms are usually considered harmful. The Baltic Sea is a relatively young brackish system composed of several subbasins, with latitudinal gradients in salinity, temperature, and nutrients, and in the central Baltic the world's largest cyanobacteria bloom is a recurring phenomenon every summer. This well-studied system is ideal for local adaptation studies^1^ and is increasingly referred to as a time machine for other coastal areas as climate effects are manifested early here^2^. During the last decades, an earlier start with a longer duration of nitrogen-fixing cyanobacteria blooms^3,4^ and reduced diatom spring blooms were observed in the Baltic Proper^5^. However, N-fixing cyanobacteria blooms have existed in the Baltic Proper for 7000 years^6,7^. In the Bothnian Sea, the cyanobacterial blooms have become regular only during the last 10–15 years^8,9^ partly due to increased phosphorous concentrations^10^. These changes in phytoplankton composition and nutrient load could have direct implications for the organisms dependent on primary producers, especially the Bothnian Sea consumers, which, unlike Baltic Proper consumers, have no history of coexistence with cyanobacteria. The decrease of diatoms in the spring bloom that sink efficiently to the seafloor has been linked to starvation of the Bothnian Sea benthic community^11^ and so-called brownification from climate induced increases in terrestrial loading from precipitation and run-off from land^12^. Moreover, increases in cyanobacterial blooms may not compensate the nutritional deficiency because cyanobacteria are low in essential lipids, i. e. polyunsaturated fatty acids and sterols^13,14^. However, most studies demonstrating the nutritional inadequacy of cyanobacteria have been conducted using lab cultures of cyanobacteria isolates which is ecologically unrealistic (e. g.,^15^).

In contrast to laboratory observations, field studies show that pelagic, littoral, and benthic consumers readily incorporate organic carbon and nitrogen from cyanobacteria in the Baltic Proper e.g.,^16–20^. Experimental studies on cyanobacteria-consumer interactions involving mixtures of field-collected cyanobacteria and phytoplankton bloom material instead of laboratory cultures are needed to increase ecological realism. Notably, the reported adverse effects of cyanobacteria e.g.,^21–23^ due to toxicity and low-quality food were challenged by more recent studies demonstrating neutral, or even positive effects of the diets supplemented with cyanobacteria e.g.,^18^. However, populations that have seldom encountered toxic cyanobacteria (such as Bothnian Sea amphipods) and those exposed regularly to this source of nutrients and bioactive compounds (such as Baltic Proper amphipods) may respond differently to cyanobacteria in the diet.

*Nodularia spumigena*, one of the dominant cyanobacteria species in the Baltic Proper, produces many bioactive compounds, including the hepatotoxin nodularin, harmful to vertebrates^24^. It has been shown that perch from lakes without cyanobacteria exhibit oxidative stress when exposed to *N. spumigena*, whereas the stress response was lower in the fish from the Baltic Proper or lakes with cyanobacteria^25^. Another stress mechanism, associated with toxic cyanobacteria, measured in fish^26^ and in clams is neurotoxicity^27^; this response is commonly measured as acetylcholinesterase (AChE) inhibition^28^. However, in freshwater lakes^29^ and the Baltic Sea^30,31^, various grazers have evolved physiological and behavioral adaptations, enhancing their ability to coexist with toxic cyanobacteria. Moreover, significant variability in growth and other fitness-related traits between Baltic copepod species feeding on *Nodularia*-rich diets has been observed and attributed to various adaptation mechanisms, including nodularin biodegraders in the host microbiome^32^.

The deposit-feeding amphipod *Monoporeia affinis* has a wide distribution in the Baltic Sea and some lakes of the region, where it is one of the most abundant species of the soft-bottom communities^33,34^. This slow-growing amphipod with a two-year life cycle is an important bentho-pelagic link through feeding on settling phytoplankton material^33,35,36^. In the Baltic Proper, no adverse effects on survival were found for *M. affinis* exposed to *N. spumigena* despite nodularin accumulation^37,38^. Another recent study compared uptake of cyanobacteria and diatoms in the Baltic proper benthos^15^, however, their interpretations that diatoms were selected over cyanobacteria were based on the assumption that increased uptake along with increased availability of the added material represented selectivity.

The main question of this study is whether the northern population of *M. affinis* is sufficiently plastic to deal with the future changes in phytoplankton, namely, decreased input of diatoms and an increase in cyanobacteria. In a common-garden experiment, two *M. affinis* populations were exposed to different feeding regimes representing various combinations of diatom and cyanobacteria contribution to the diet to address this question. One of the populations originated from the northernmost part of the Bothnian Sea (BoS), where cyanobacterial blooms appeared only recently, and the other population originated from the Baltic Proper (BP), where large cyanobacterial blooms are regular phenomena during the last 7000 years^6^.

We expected BoS amphipods to be dietary specialists adapted to the diatoms as a single food source, with a small trophic niche and low potential for plasticity. In contrast, the more heterogeneous food environment in the BP, with spring blooms of diatoms and summer blooms to cyanobacteria, should result in *M. affinis* populations being adapted for cyanobacteria, having more generalist feeding habits and potential for niche expansion when exposed to mixed foods. More specifically, we hypothesized that:

- [1] BoS amphipods would have the highest growth rate and body condition when offered a surplus of diatoms because they are specialized in utilizing this food source.
- [2] BP amphipods would grow better than BoS amphipods in the mixed diatom/cyanobacteria diet treatments, especially when the diatom contribution is low. Moreover, the growth and body condition of BP amphipods would respond positively to the cyanobacteria addition to the diet compared to diatom mono-diet because cyanobacteria have complementary nutrients.
- [3] BP amphipods would have a larger feeding niche in the mixed-diet treatments reflecting higher diversity of the food sources. In contrast, BoS amphipods would selectively assimilate diatom-derived nutrients resulting in a small niche size.
- [4] The assimilation of cyanobacteria-derived nutrients will coincide with higher neurotoxicity and nodularin levels in both populations.

As tracers of assimilation of bloom material we take advantage of the distinct isotope signatures (especially for δ^15^N) of field-collected cyanobacteria (depleted ^15^N) and diatoms (enriched ^15^N due to targeted collection in a bay with influence from N from a sewage treatment plant^39^, and we quantify the trophic niche using the isotope niche concept^40–42^.

## Methods

### Collection of sediment, amphipods, and phytoplankton

**Figure 1:**
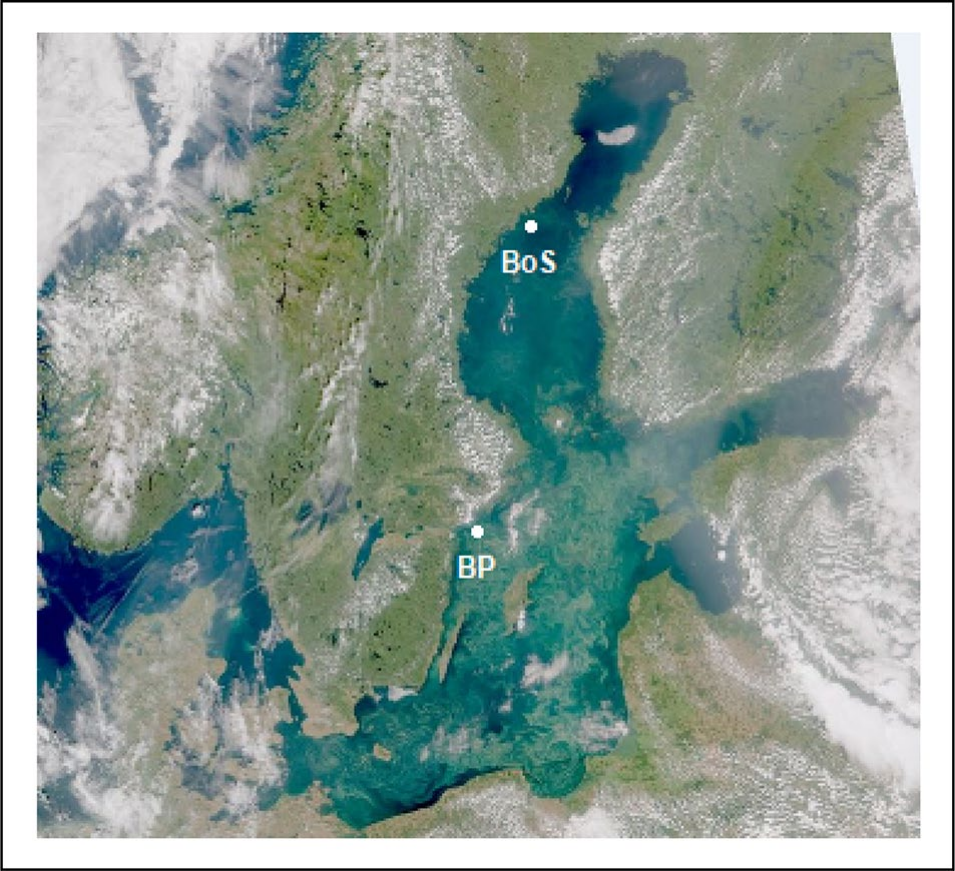
Satellite view of the Baltic Sea on July 25, 2019 (NOAA: Satellite SuomiNPP; data processed by SMHI), showing cyanobacterial blooms covering a great part of the Baltic Proper. White dots represent collection sites for *Monoporeia affinis* used in the common-garden experiment in the Baltic Proper (BP: stn. Grund utsjö) and northern Bothnian Sea (BoS: stn. N21). The experimental sediment, diatoms, and cyanobacteria were collected close to the BP station (see text for differences in characteristics among sediments).

The experimental sediment was collected in late March (2017), before the onset of the annual spring bloom, in the Baltic Proper (BP), (58° 43´41´´N, 17° 41´ 00.72´´E; Fig. 1), with a benthic sled from 30 m depth^43^. The organic carbon content of this sediment was 2%. The sediment was stored in a cold (2°), dark room with filtered brackish water and aeration. Two weeks before the experiment, the sediment was sieved through a 1-mm sieve to remove macrofauna.

Amphipods were collected in the Baltic Proper: station Grund Utsjö (45 m depth, organic carbon content of 0.9%) and the Bothnian Sea: station N21 (62 m depth, org C content 0.5%) with a benthic sled^43^ before the start of the spring bloom (late March in the BP and early June in the BoS, both in 2017 (Fig. 1). Amphipods were carefully sieved from the sediment (mesh size 1 mm), transported to the Askö laboratory, Stockholm University, and incubated in darkness with some sieved sediment from the respective stations at 4 °C and aeration until the start of the experiment.

Diatoms dominated by *Thalassiosira baltica* (~ 95% of phytoplankton by visual inspection; Dr. Helena Höglander, Stockholm University, pers. comm.) were collected at the peak of the spring bloom (March 30th, 2017) at station H4 (58° 59′ 02 N, 17° 43′ 50 E) in the outer Himmerfjärden Bay (close to a monitoring station Grund Utsjö) and stored in darkness at 1 °C with aeration until the start of the experiment (cells were visually inspected before the experiment to confirm that they were were not lysed). Summer bloom material, composed mainly of the nitrogen-fixing cyanobacterium *Nodularia spumigena* (97% of the total biovolume), was collected in July 2006^44^ and stored frozen (− 20 °C). *N. spumigena* filaments do not break during freezing or thawing, and nodularin is also resistant to storage^45^. The δ^15^N in this stock was − 2‰^44^ and hence different from the diatom signal (15‰), enabling its tracing in consumers assimilating the cyanobacterial nitrogen^17^.

### Experimental design

The 5-week experiment was conducted in June 2017, in a thermo-constant room of Askö Laboratory, at 3 °C and in darkness, i.e., the conditions resembling in situ environment for this species. Amphipods with similar body sizes from both populations were allocated to microcosms grouped by five treatments; 15 amphipods/microcosm and 7 replicates/treatment were used. Each microcosm, a 1 L plastic jar with a 4-cm layer of the sieved sediment and 15 cm of the overlying water, was supplied with gentle air bubbling, and all microcosms were placed in random order in the experimental room. Subsamples of the concentrated diatom and cyanobacteria material were analysed for carbon and nitrogen content by adding known volumes to pre-combusted GFF filters followed by elemental analyses at the accredited laboratory of the Center for Physical Science and Technology (Vilnius, Lithuania). By dry mass, the C content of diatoms and cyanobacteria was 21% and 23%, respectively, and N content was 2% and 2.9%, respectively.

**Figure 2:**
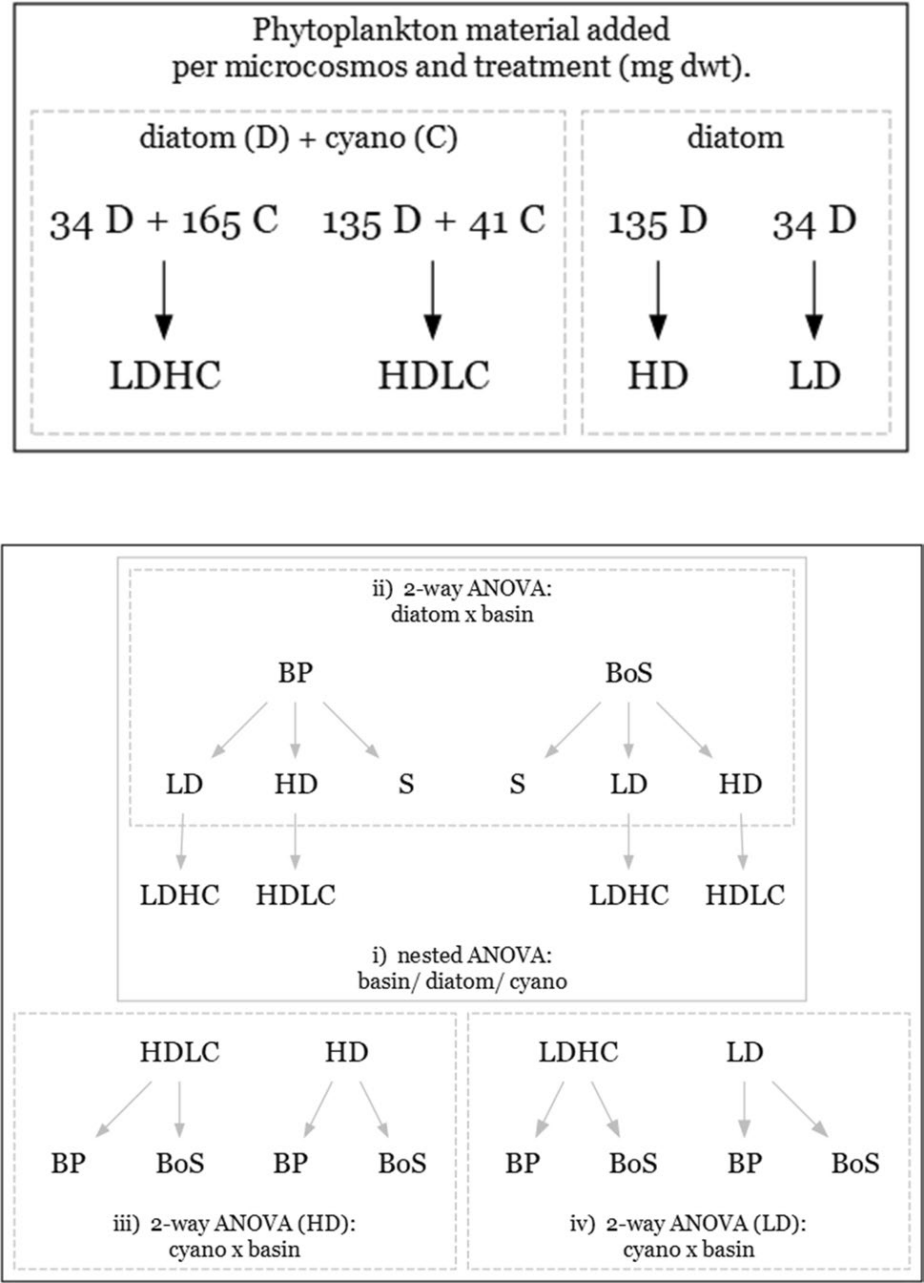
Summary of the experimental design and statistical approaches to test the effect of different diet regimes (*LD* low diatom, *HD* high diatom, *LDHC* low diatom high cyanobacteria, *HDLC* high diatom low cyanobacteria, *S* sediment only) on amphipods from the BP and BoS basins. The phytoplankton material added per microcosm in the experimental treatments and expressed as dry mass (mg dwt) of the diatoms and cyanobacteria for high and low levels of each food type. See details on statistical tests in the text.

The feeding treatments (Fig. 2) represented five plausible scenarios for resource availability: (i) high diatom quantity (HD) would correspond to a strong diatom bloom in spring and no measurable cyanobacteria input during summer (i.e., the historical regime in BoS; note that the spring bloom material remains in the sediment and fuels the benthos for months^46^; (ii) low diatom quantity (LD) would correspond to a weak diatom bloom in spring and no measurable cyanobacteria input during summer, (iii) high diatom quantity and low cyanobacteria quantity (HDLC) would correspond to a strong diatom bloom in spring and some cyanobacteria sedimentation during summer; (iv) low diatom quantity and high cyanobacteria quantity (LDHC) would represent the forecasted primary production regime in both basins when a weak diatom bloom is predicted to be combined with a heavy cyanobacteria bloom; and (v) control with no added phytoplankton (sediment only, S). The target amount of the food added to each microcosm for HD and LD (Fig. 2) were equivalent to the average levels observed in the northern Baltic Sea during high (4.9 g C m^−2^) and low (1.0 g C m^−2^) blooms, respectively^47,48^.

The experiment was started with the food addition and terminated 5 weeks later by sieving the sediment, counting surviving individuals, and leaving them in filtered seawater for 24 h to empty their guts. After that, they were frozen at − 80 °C individually for subsequent analyses.

### Endpoints

The following endpoints were measured in the same individuals (3 individuals per microcosm): individual body mass (dry weight; a measure of somatic growth), carbon-to-nitrogen ratio (C:N ratio, a proxy for lipid content in *M. affinis*^36^, and stable isotope composition of carbon and nitrogen (δ^13^C and δ^15^N). Another 3 individuals per microcosm were used for protein concentration and AChE activity (the latter indicative of neurotoxicity from e.g. cyanotoxins) measurements. Animals sampled at the start of the experiment (hereafter referred to as initials) were also analysed for body mass, stable isotope signature, elemental analysis, and protein content. Additionally, nodularin/microcystin levels were measured in the amphipods from the LDHC and control treatments, the sediment collected in BoS and BP, and the frozen cyanobacteria. For this analysis, pooled samples were used as described below.

### Stable isotope and elemental composition

The amphipods (n = 197) and subsamples of sediment (homogenized separately using a mortar and pestle), the cyanobacteria, and the diatom bloom materials were analysed for bulk δ^15^N and δ^13^C at the Center for Physical Science and Technology, Vilnius, Lithuania. A Flash EA 1112 Series Elemental Analyzer connected via a Conflo III to a Delta V Advantage isotope ratio mass spectrometer (all Thermo Finnigan, Bremen, Germany) was used for the analysis. Ratios of ^14^N:^15^N and ^12^C:^13^C were expressed in permille deviations relative to the lab standards and back-calculated to international standards, atmospheric air (N), and Pee Dee Belemnite (C). An internal reference (fish muscle tissue) was analysed every 10 samples. Overall analytical precision was better than ± 0.15‰ for δ^15^N and ± 0.10‰ for δ^13^C values.. Note that the isotope approach aims at tracing the disctinct signal (similar to artificially enriched algae, e.g.,^44^ not to perform a mixing model since enrichment factors are unknown for this slow growing species and generally for benthic species deviating in the carbon trophic enrichment factor^49^.

### Isotopic niche analysis

The variation in isotope composition among individuals in a population can be used as a proxy of the trophic niche^40,42^, hereafter referred to as the “isotopic niche”. A larger isotope niche indicates a substantial diet variation among the individuals, whereas a narrow niche suggests a more uniform diet in the population. A large isotope niche may however also indicate higher intrapopulation variability in growth or physiological status since the diet-consumer fractionation depends on metabolic processes^50–52^. Hence, it is advantageous to know the feeding biology of the studied species, which is the case for *M. affinis*, including fractionation responses to suboptimal nutrition, when interpreting their isotope values^44,51,53^.

For the analysis of the isotopic niche, we used the total area of the convex hull and the standard ellipse area (TA and SEAc, respectively); these are the primary niche size descriptors, with SEAc being the more robust measure^41,42^. In addition, the maximum range in δ^15^N and δ^13^C values, respectively (NR and CR; the trophic length of the population, and the diversity of basal resources, respectively) were used.

### Sample preparation for protein and AChE analyses

To each cryotube (2 ml) containing one amphipod, acid-washed glass beads (212–300 µm; Sigma Aldrich, Germany) and 180 µL potassium phosphate buffer (0.1 M, pH 7.2) were added. The cryotube was run in a bead beater Fast-Prep Fp120 (Thermo Savant, USA) at 5.5 m/s for 20 s and cooled in an ice bath for 10 s; this cycle was repeated three times. After the bead beating, the cryotubes were centrifuged in 4 °C at 3300 × *g* for 5 min using an Allegra X-30r Centrifuge (Beckman and Coulter, USA). The supernatant for the protein assay (65 µL) and AChE analyses (65 µL) was cooled on dry ice and stored at − 80 °C.

### Protein assay

Pierce BCA Assay kit (catalog# 23227, Thermo Scientific, USA) for microplate procedure, with bovine serum albumin (BSA; 20–2000 µL/mL) as a standard, was used for measuring protein content in the amphipods. Into each well of a 96-well microplate with a clear flat bottom (Corning) kept on ice, 10 µL plasma protein binding (PPB) were pipetted followed by 15 µL test sample. The following program was used: shaking for 30 s, 37 °C for 2 h, and cooling to room temperature. The absorbance was measured at 540 nm with Hindex Senese Microplate Reader (Hindex, Oy, Finland). All samples were analyzed in duplicates.

### AChE analysis

The AChE activity was measured in 252 amphipods following the colorimetric absorbance method ^54,55^, with acetylthiocholine iodine (AcSCh) as a substrate, and 5,5′-dithiobis(2-nitrobenzoic acid) (DTNB) as the reagent. The sample protein concentration was adjusted to 0.5 mg/L using PPB as a diluent. The microplate was shaken for 2 min, and absorbance was measured at 405 nm every 2 min for 10 cycles. This measurement was performed at 25 °C using the same microplate reader as for the protein assay. The AChE activity was expressed in nmol of the substrate per mg protein and min (nmol/mg/min); see Eq. (1).

1 $$AChE\ activity= \frac{\Delta A\cdot F}{\varepsilon \cdot l\cdot t\cdot c}$$

where, ΔA represents the change in the absorbance at 405 nm and F is the ratio between the total volume and the sample volume, ε is the extinction coefficient for DTNB, l is lightpath (microplate well depth), t time, and c is protein concentration in mg/mL.

### Nodularin concentration in cyanobacteria, sediment, and amphipods

The cyanotoxin concentration was analysed using ELISA microcystin plate kit (ADDA SAES, Abraxis Laboratory) and a nodularin standard. The assay quantifies both nodularins and microcystins; however, as 97% of the sample material consisted of *N.spumigena*, we consider our measurements to represent nodularin. The lyophilized samples of amphipods (LDHC and the control treatments of each population), the *N. spumigena* bloom material, and the sediment from BoS and BP were homogenised and extracted with 2 ml methanol (100% HPLC quality) in glass tubes. To have sufficient biomass for nodularin detection in the amphipod samples, we pooled 4–5 individuals to a total dry mass of ~ 6 mg. After that, the samples were shaken twice in an ultrasonic ice bath (Cole Parmer 8891) for 60 min with a 24 h resting period at 4 °C. To each tube, 0.5 ml MilliQ water were added, and the samples were centrifuged (Sorvall 16R, Thermo Fisher Scientific; 20 min at 10,000 × *g*). Finally, the supernatant was reduced to 0.5 ml with a SpeedVac concentrator (Savant SPD1010, Thermo Fisher Scientific) at 45 °C, and the samples were stored at − 20 °C until the ELISA assay conducted according to the manufacturer’s instructions.

### Water chemistry

Static exposure was used in the experiment, and de-ionized water was added to compensate for the evaporation loss. Upon termination of the experiment, we measured inorganic dissolved nutrients to rule out the adverse effects of elevated concentrations of ammonium and nitrates on the amphipod survival and growth. Water was collected from three randomly chosen replicates per treatment and population using a sterile syringe, filtered through a 0.2 µm Millipore, and frozen immediately. In these samples, ammonium and nitrate concentrations were analysed by the accredited laboratory at the Department of Ecology, Environment and Plant Sciences, Stockholm University (see Supplementary Information, Fig S3).

### Statistical analysis

We used a nested ANOVA design (Fig. 2 panel i) to investigate the effects of population origin (*basin*; 2 levels: Baltic Proper and Bothnian Sea); diatom addition (*diatoms*; 3 levels: none, low and high), and cyanobacteria/ diatom mixed diet (*cyano*; 2 levels: high proportion and low proportion) on most of the measured endpoints (Fig. 2). All three factors (*basin*, *diatom,* and *cyano*) were fixed since none of them were randomly chosen and instead represent real diet scenarios in the Baltic Proper^44^. The microcosms were used as replicates, with individual measurements within each microcosm (n = 3) treated as technical replicates and averaged before statistical analyses. An initial analysis using LMM was performed prior to ANOVA but the variance among individuals within replicates was so low that ANOVA was deemed more parsimonious.

Thereafter several ANOVAs were performed to test hypotheses 1, 2 and 4 as shown in Fig. 2 (panels ii–iv). A two-way ANOVA was used to test the effects of the basin and diatom addition and high/low (three levels; S (control), LD (low) and HD (high Fig. 2, panel ii) on the various response parameters (Hypothesis 1). The control treatment (S) was omitted when testing uptake of diatoms since starvation-induced inflated δ^15^N values in this treatment without added food may confound the diatom-assimilation signal (i.e. *M. affinis* can not grow from aged sediment only, this will inflate its isotope composition^51^). A two-way ANOVA was also used to test the effect of cyanobacteria addition to high- (panel iii) and low- (panel iv) diatom diet (Hypothesis 2 and 4). When the interaction term was found non-significant, only results from the nested incomplete design ANOVA (i) are presented for simplicity (Hypotheses 2 and 4) and 2-way ANOVA results are presented in Supplementary Information.

Nodularin concentrations in the amphipods from LDHC and control treatments were tested with two-way ANOVA to evaluate the effects of cyanobacteria addition, Basin, and the interaction (Hypothesis 4).

A Bayesian framework implemented in the SIBER R package^42^ was applied to evaluate the treatment and basin effects on the isotopic niche size in the experimental animals. A Shapiro–Wilk test was used to test the multivariate normal distribution assumption with the R package ‘*mvnormtest*’^56^. Bayesian estimates of the standard ellipse (SEAb) were used for pairwise comparisons between the mixed and mono-diet treatments and the controls (Hypothesis 3).

Basic statistical tests (Unpaired *t* test and Wilcoxon signed-rank test, for normal and not normally distributed data, respectively) were performed to compare initial conditions between the basins for each endpoint. To check that variability in survival did not invalidate our hypothesis testing, we also performed tests to see whether survival differed between populations (Wilcoxon Rank Test) and within populations among treatments (Kruskal–Wallis test). All data were explored for potential outliers before the analysis; only true outliers above/below the 75th or 25th percentile were removed (six outliers in total out of 449 values). The AChE activity values were log (x + 1) transformed. In all figures, data are presented as untransformed mean ± SE values, except survival (median with max and min) and the AChE activity (geometric mean, GM, with 95% confidence interval), where the data were right-skewed. Homogeneity of variance was visually inspected and tested with Bartlett´s test. All statistical analyses were performed using the R software environment 4.1.0^57^.

## Results

### Survival

Survival was high, with average values varying between 86 and 100%. However, two replicates in the LD treatment for the BP population had only 40 and 55% survival (Fig. S1, Supplementary Information). Kruskal–Wallis test showed no treatment effect in each population (BP: H(4) = 1.916, p > 0.7; BoS: H(4) = 7.799, p > 0.09). Similarly, there was no significant difference in mortality between the populations (Wilcoxon Rank Test; W = 570, p > 0.6).

### Stable isotope composition in food sources and consumers

**Figure 3:**
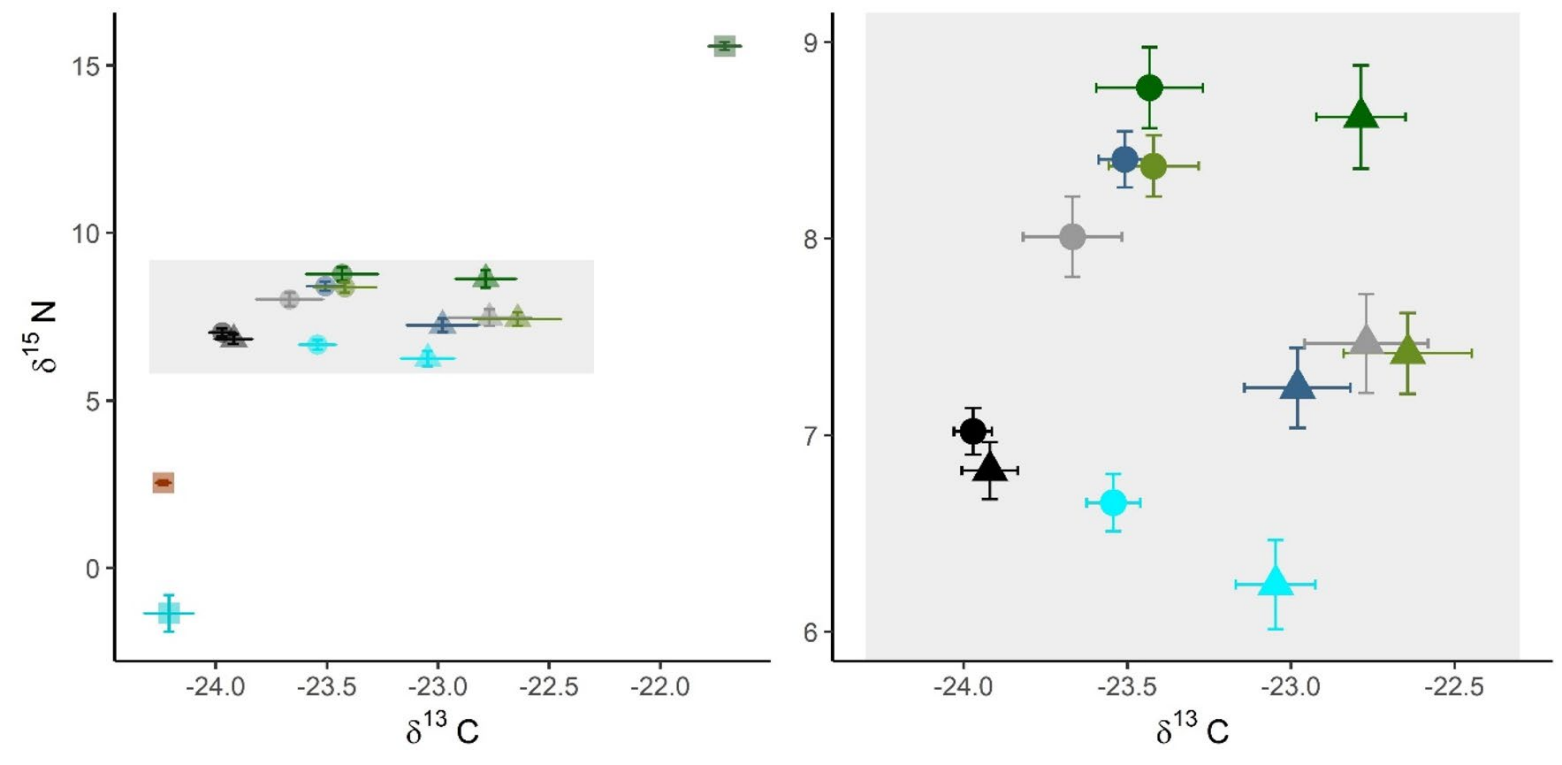
Stable isotope bi-plot of δ^15^N and δ^13^C values in the different components of the experimental system. Left panel: the food sources in the experimental microcosms (squares), including sediment (brown), diatoms (dark green), and cyanobacteria (turquoise), and the test animals (shaded field). Right panel: a zoom-in of the shaded field from the left panel showing the signatures of the amphipods originated from the BP (triangles) and BoS (circles) populations. The colour coding: Initials (black) and the treatments: Control (grey), HD (green), HDLC (blue), LD (olive green), LDHC (turquoise). The data are shown as group means with SE as error bars.

As expected, the food sources (diatoms and cyanobacteria) differed clearly in their δ^15^N and δ^13^C values (Fig. 3), demonstrating their utility as diet tracers in this experiment.

### δ^15^N in amphipods

There was no significant *basin* effect on the initial amphipod δ^15^N values (BP: 6.8 ± 0.7 and BoS: 7.0 ± 0.5; t = 0.901, df = 32, p > 0.3). In the course of the experiment, an increase in the δ^15^N values was observed in the amphipods without any food addition (S treatment) as indicated by significantly higher values in the controls than in the corresponding initials (0.7 and 1.0 ‰ increase for BP and BoS, respectively (Table S1, Supplementary Information). When testing the effects of the increasing addition of ^15^ N-enriched diatoms (Fig. 3; *ii* panel) on the δ^15^N values in amphipods, we found a significant *basin* effect with higher values in the BoS than in BP. Moreover, the *diatom* effect was also significant (HD > LD). The interaction effect (*diatom* × *basin*) was marginally significant (p < 0.07; Table 1), with the difference between HD and LD being more pronounced for BP than BoS amphipods (Fig. 3). When testing the effects of the cyanobacteria addition to the diatom diet, the outcome depended on the cyanobacterial contribution to the diet. In the diet with high cyanobacteria contribution (Fig. 2, iv panel), both *cyano* and *basin* effects were significant, as indicated by the lower δ^15^N values in LDHC than LD treatments and BP than BoS populations (Table 1a). At the low cyanobacteria contribution (Fig. 2, iii panel), a significant *cyano* × *basin* interaction effect was found (Table 1b), with a significant effect of cyanobacteria addition (HDLC < HD) for the BP, but not for the BoS amphipods.

**Table 1:** Results from two-way ANOVA model, testing the effect of diatoms and cyanobacteria in the diet (a: *diatom*; b and c: *cyano*) and *basin* (BoS vs. BP) as main factors and their interactions (*basin* × *diatom* or *basin* × *cyano*) on δ^15^N values in amphipods. See Fig. 3.

Variables	SS	df	MS	F	p-value
**δ**^**15**^**N**					
(a) Increased diatom input (LD and HD)					
* Basin*	4.528	1	4.528	10.294	**0.004**
* Diatom*	1.914	1	1.914	4.350	**0.048**
* Basin* × *diatom*	1.681	1	1.681	3.821	*0.062*
Residuals	10.558	24	0.440		
(b) High proportion of cyanobacteria (LDHC vs. LD)					
* Cyano*	15.156	1	15.156	53.364	**0.000**
* Basin*	3.789	1	3.789	13.341	**0.001**
* Cyano* × *basin*	0.538	1	0.538	1.893	0.181
Residuals	6.816	24	0.284		
(c) Low proportion of cyanobacteria (HDLC vs. HD)					
* Cyano*	6.166	1	6.166	13.098	**0.001**
* Basin*	2.413	1	2.413	5.126	**0.032**
* Cyano* × *basin*	2.151	1	2.151	4.568	**0.042**
Residuals	11.299	24	0.47		

Significant values are in bold and marginally significant values (< 0.1) in italics.

### δ^13^C in amphipods

There was no significant *basin* effect on the initial amphipod δ^13^C values (BP: − 23.9, BoS = − 23.9; W = 140.5, p > 0.9), see Fig. 3. Nested ANOVA on experimental amphipods (Fig. 2, i panel) showed a significant *basin* effect, with higher values for BoS amphipods; this effect was also significant in the mono-diets and in the pairwise comparisons (mixed vs. mono-diatom diets, two-way ANOVA; Table S2a–c, Supplementary Information). Only at high cyanobacteria contribution (LDHC vs. LD), there was a marginally significant *cyano* effect (Table S2b, Supplementary Information).

### Individual body mass and growth

There was a marginally significant basin effect on the initial amphipods body mass (t = 1.87, df = 32, p < 0.07, BoS: 0.91 ± 0.16 mg and BP: 0.76 ± 0.24 mg, mean ± SD). Amphipods did not increase their body mass from the initials in the control treatment (4 vs. 5% average increase in BoS and BP populations, respectively; Table S3, Supplementary Information). The nested ANOVA showed non-significant differences in growth (body mass change relative to the initials) of the experimental amphipods between *basin* or due to the diatom addition (contrary to Hypothesis 1), while cyanobacteria addition had a significant positive effect (Hypothesis 2; Table 2, Fig. 3). According to the two-way ANOVA, the positive effect of *cyano* on growth was significant (p < 0.05) for the treatments with a high proportion of *N. spumigena* (Fig. 2, iv panel) but not for the low proportion treatments (Fig. 2, iii panel; Table S4b–c, Supplementary Information). Despite the non-significant differences in the latter comparison, the BP amphipods’ body mass showed an average increase of 23% compared to 13% by BoS amphipods (Table S3, Supplementary Information).

**Table 2:** Nested ANOVA testing effect of the mixed diet (*cyano*; high/low cyanobacteria proportion), the diatom addition (*diatom*; *HD* high diatoms, *LD* low diatoms, *S* sediment only), and amphipod population origin (*basin*) on the response variables: δ^13^C values, C:N ratio, body mass change relative to the initials (Growth) and Acetylcholinesterase (AChE) activity; see also Figs. 3 and 4.

Effect	SS	df	MS	F	p-value
**δ**^**13**^**C**					
*Basin*	7.96	1	7.96	59.934	**0.000**
*Basin:diatom*	0.598	4	0.15	1.127	0.353
*Basin:diatom:cyano*	0.708	4	0.177	1.334	0.268
Residuals	7.836	59	0.133		
**Growth**					
*Basin*	0	1	0	0.001	0.974
*Basin:diatom*	0.092	4	0.023	1.132	0.350
*Basin:diatom:cyano*	0.211	4	0.053	2.606	**0.045**
Residuals	1.194	59	0.020		
**C:N ratio**					
*Basin*	13.821	1	13.821	50.446	**0.000**
*Basin:diatom*	0.978	4	0.245	0.893	0.474
*Basin:diatom:cyano*	4.665	4	1.166	4.257	**0.004**
Residuals	16.165	59	0.274		
**AChE activity**					
*Basins*	0.849	1	0.849	6.386	**0.014**
*Basins:diatom*	0.192	4	0.048	0.361	0.835
*Basins:diatom:cyano*	0.609	4	0.152	1.146	0.344
Residuals	7.980	60	0.133	7.980	

Significant values are in bold.

### C:N ratio

**Figure 4:**
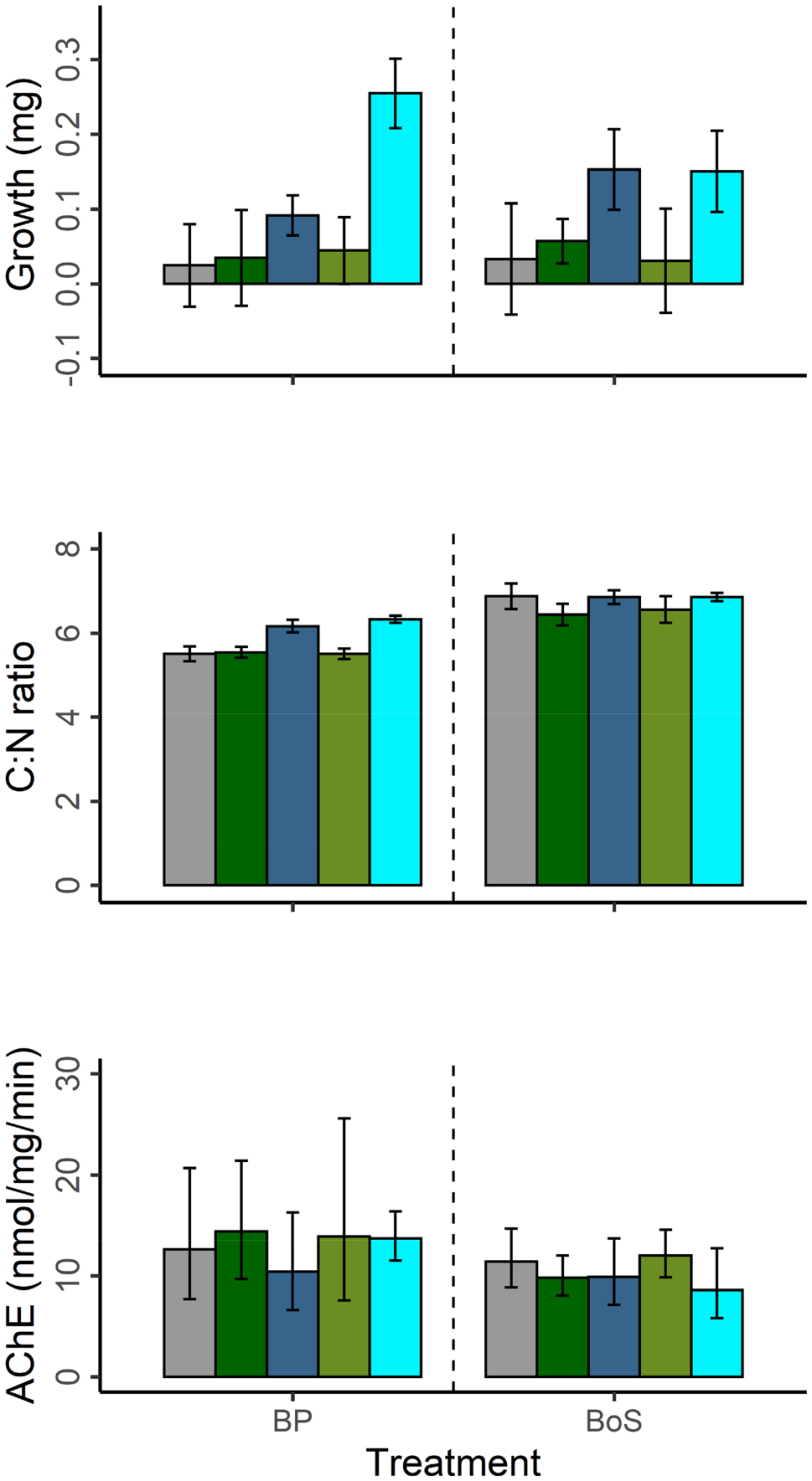
Body condition parameters (Growth and C:N ratio) and Acetylcholinesterase (AChE) activity for amphipods from each population and diet treatment. The diets include supplementation of the control sediment (S) with Low and High Diatom (LD, HD), and a combination of diatoms with low and high cyanobacteria (LDHC, HDLC). Colour represents each treatment: Control (grey), HD (green), HDLC (blue), LD (olive green), LDHC (turquoise). Values are mean ± SE for body conditions parameters and geometric mean [95% CI] for AChE activity. See Table 2 for statistical comparisons and Table S3, Supplementary Information: with absolute differences for the treatments under comparison.

There was a significant basin effect on the initial amphipods C:N ratio, with higher values for BP amphipods (BP: 7.65 ± 0.94, BoS: 7.03 ± 0.54; t = − 2.47, df = 29, p < 0.02). During the experiment, the control animals from BP showed a significant 27% decrease (t = − 6.11, df = 30, p < 0.001) in the C:N ratio compared to the initials, while in the BoS amphipods, the C:N ratio was unchanged (Table S3, Supplementary Information). Results from the nested ANOVA showed a significant *basin* effect*,* with higher values for BoS amphipods. In addition, there was a significant positive *cyano* effect on the C:N ratios (Table 2, Fig. 4) with a greater percentage increase compared to the diatom only-diet for BP than for BoS amphipods (Fig. 4, Table S3, Supplementary Information).

### AChE inhibition

The BP and BoS populations showed no significant differences in AChE activity (nmol/mg/min) between the controls (BoS: 11.4 [8.8, 14.7], BP: 12.6 [7.7, 20.7], geometric mean [95% CI]; *t* test, t = − 0.348 p > 0.7). Nested ANOVA demonstrated significant differences between the basins*,* with lower AChE activity for BoS than BP amphipods (Fig. 4, Table 2). Both mixed treatments (cyanobacteria, diatoms) showed lower AChE activity compared diatom only treatments (Table S5, Supplementary Information).

### Nodularin concentrations

The nodularin concentration in the *N. spumigena* bloom material used for the experiment was 360 µg/g. The nodularin concentration in the initial sediments differed between the basins, with higher values in BP than BoS (3.6 and below the detection limit of 0.7 ng/g, respectively) which was in agreement with the significant difference for experimental amphipods between basins (Table S7: Supplementary Information, Fig. S2). There was a marginally significant (p > 0.082) treatment effect with a higher nodularin concentration in the exposed amphipods than in control amphipods (on average; 220 and 118 ng/g, respectively).

### Isotope niche as a population-level response

**Figure 5:**
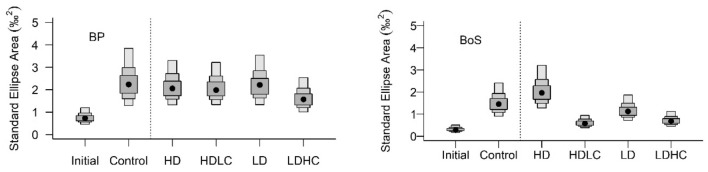
Density box-plot of Bayesian estimates of Standard Ellipse Area (SEAb) for each treatment and population with initial and control amphipods to the left of the dotted vertical line. Black dots indicate the SEAb with 50, 75 and 95% credible intervals produced from 10^5^ Bayesian iterations of SEA (SEAb).

The niche size (SEAb) for the initials was larger in the BP population than in the BoS population by 40%. In the controls, SEAb increased relative to the initial size by 30% for BP and 20% for the BoS amphipods (Table 3, Fig. 5). BoS amphipods showed a more variable pattern in the niche size among treatments compared to the BP amphipods which showed no major change. Mixed-diet treatments (HDLC, LDHC) in BoS amphipods showed niche compression relative to the control, and the HDLC treatment showed a significant compression of 70% relative to the HD treatment (Table 3). Bayesian Layman niche metrics in initial amphipods showed higher values for all metrics for BoS than for BP amphipods (Table S8, Supplementary Information). When niche metrics in mixed treatments were compared to the diatom treatments, the BoS population generally showed expansion and the BP population compression (Table S8, Supplementary Information).

**Table 3:** Pairwise comparisons of the Bayesian standard ellipses area (SEA_B_) between the treatments testing effects of diatom and cyanobacteria addition to the control sediment for each basin (BP vs. BoS).

Treatment comparisons	Probability, BP	Difference in SEA_B_ (%)	Probability, BoS	Difference in SEA_B_ (%)
$\mathrm{HD vs}.\mathrm{ HDLC}$	0.52	− 2	1	**− 70**
$\mathrm{LD vs}.\mathrm{ LDHC}$	0.82	− 27	0.93	− 40
$\mathrm{Control vs}.\mathrm{ HDLC}$	0.61	− 10	0.99	**− 60**
$\mathrm{Control vs}.\mathrm{ HD}$	0.59	− 8	0.18	+ 34
$\mathrm{Control vs}.\mathrm{ LDHC}$	0.84	− 30	0.98	**− 53**
$\mathrm{Control vs}.\mathrm{ LD}$	0.54	− 4	0.22	− 22
$\mathrm{Control vs}.\mathrm{ Initial}$	0.99	**32**	1	**21**

The probability of the different SEA_B_ values (fold change, increase/decrease) was considered significant when > 0.95 (% Difference is shown in bold).

## Discussion

Our results integrate sub-cellular to population-level responses and provide a mechanistic understanding for the observed feeding responses in amphipods exposed to the various diet regimes. Such understanding is critical for predicting how key species and hence entire ecosystems will respond to the changing climate. Our initial prediction of the northern population being a dietary specialist was supported by the smaller isotope niche size in the field-collected amphipods, relative to that in the Baltic Proper.

The Bothnian Sea amphipods (specialist population) had similar incorporation of diatom-derived nitrogen at low (LD) and high (HD) diatom availability, while those from the Baltic Proper incorporated more when the diatoms were provided at a high amount. This indicates a higher efficiency of consumption and/ or assimilation of the diatom material by the BoS animals, further supporting Hypothesis (1) that this population is specialized to utilize diatom-based food. Population-specific feeding response is further supported by the results from the mixed-diet treatment dominated by diatoms (HDLC): the BoS population showed δ^15^N values nearly as high as in the HD treatment, indicating similar reliance on the diatoms and low consumption/assimilation of cyanobacteria. In contrast, the BP population actively utilized nitrogen originating from the cyanobacteria, as indicated by significantly lower δ^15^N values in HDLC than HD treatment. When the cyanobacteria were plentiful in the food mixture, the cyanobacteria consumption/assimilation was significant regardless of the population origin (in contrast to Hypothesis (1), as indicated by the lower δ^15^N values in the LDHC treatment compared to initials. Alternative explanations to a high assimilation of cyanobacterial derived nitrogen could be a proportionally larger consumtion of sediment (which had a lower δ^15^N value than diatoms but not as low as cyanobacteria, Fig. 3) or that the trophic discrimination factor of ^15^N is lower during rapid growth which would confound the dietary signal^50^. Considering the nodularin accumulation results (see below), it is however clear that both populations fed on the cyanobacterial material.

Along with the considerable assimilation of diazotrophic N in the LDHC treatments in both populations, although to a lesser extent in the HDLC treatment for the BoS population, growth and body condition proxies (C:N ratio) showed higher values in the mixed diets compared to the respective diatom treatments (Fig. 4, Table 2). Thus, a positive effect of cyanobacteria addition was observed in both populations, albeit stronger for the BP population. Hence, our findings support other experimental studies demonstrating the beneficial effects of cyanobacteria as a nutritional complement for invertebrates in the Baltic proper e.g.,^16,18^. Indeed, the use of field-collected cyanobacteria increases the ecological realism by providing the consumers with other nutrients not present in cyanobacteria cultures (e.g., associated microorganisms), which would improve their nutritional quality. The cyanobacterial material used in this experiment was analysed for fatty acid (FA) composition and even though the proportion of essential FAs was low compared to diatoms, the total amount of polyunsaturated FAs (including precursors to essential FAs) was actually higher than in diatoms^58^. In agreement with this finding, but in disagreement with Hypotheses 1 and 2, the growth and body condition in the BoS amphipods feeding on the diatom/cyanobacteria mixtures were similar or higher compared to the high diatom treatment. It is worth poiting out, however, that the treatments with cyanobacteria also received more food in total. Although the difference is small between the HDLC and LCHD treatment (within 20% difference in total biomass added), both these mixed diets had in average 70% higher OM addition compared to the HD treatment, highlighting that comparisons in growth output between mixed diet treatments and the HD treatment should be carefully interpreted. Regardless, the main results; that also the northern population feed on cyanobacteria without fitness penalties hold true.

In *Daphnia*^59^ and other invertebrates (e.g.,^60^), activation of digestive enzymes is dependent on the quantity and quality of the food; facilitating digestion of novel resources. Thus, a possible explanation for low cyanobacterial utilization in the HDLC treatment by BoS amphipods could be that the digestive enzymes breaking down cyanobacteria are produced in low quantities, either because of the low cyanobacteria availability or due to active avoidance. In the LDHC treatment, the low quantities of the diatoms resulted in the higher encounter and consumption/assimilation of cyanobacteria, possibly enhancing the production and activities of the digestive enzymes for this food, as shown for *Daphnia* exposed to diatoms or cyanobacteria^61,62^.

Amphipods from both basins accumulated nodularin in their bodies, supporting a previous study on *M. affinis* exposed to the same cyanobacterial material (although in a larger quantity^38^). Interestingly, the nodularin levels measured in the amphipods from the control treatments were similar to what was found in the clam *Macoma balthica* in the Gulf of Finland after the cyanobacteria bloom^27,63^. As the sediment nodularin concentrations in the control treatment (originating from the BP) were in the range of field measurements (2.3–0.18 ng/g) before cyanobacterial bloom^45^, our control can therefore not be considered a true control regarding neurotoxic exposure. However, at high cyanobacteria proportion in the diet, the highest average nodularin levels coincided with the most considerable AChE inhibition relative to the diatom treatment (21%) and the control (17%) found in the BoS amphipods (supporting Hypothesis 4). Similar AChE inhibition (19%) in *L. balthica* exposed to *N. spumigena* resulted in behavioral changes^27^. In BP amphipods exposed to a high-cyanobacteria diet, the low AChE inhibition (only 5%) may indicate adaptation to these cyanobacteria due to the long history of coexistence (in contrast to Hypothesis 4), similar to what has been shown for perch (*Perca fluviatilis*) from geographic areas with and without cyanobacterial blooms^25^. Moreover, when provided with a sufficient amount of alternative food (diatoms), which reflects the field situation, these amphipods might compensate for the energetic cost of detoxification.

Results from this study have implications for the overall importance of feeding plasticity during environmental change. The high feeding plasticity in the BoS population, as assessed from the large isotopic niche variability among treatments (Fig. 5) suggests that these amphipods may not be as strictly specialized in their feeding as expected. The BoS population showed significant niche expansion in the two diatom treatments (LD and HD) compared to the mixed treatments and the controls. The niche expansion in BoS amphipods may partly be due to between-individual variability in feeding preferences^64^, e.g., some individuals feed on diatoms and others on aged organic matter in the sediment. This possibility was partially supported by the higher variation in body conditions of amphipods in diatom than in the mixed treatments. Accordingly, larger variability in body condition was present already in the initials of the BoS population, which could translate into isotope niche expansion due to differences in growth and metabolic status. An alternative but non-exclusive hypothesis is that fresh phytoplankton in high quantities (LDHC > HDLC > HD > LD) promoted more uniform feeding within a population and a higher body condition due to a favorable relationship between time searching for food and energy gain^65^.

Reproductive success in *M. affinis* is affected positively by food quality and quantity^66^. During suboptimal feeding conditions, the lipid accumulation is hampered, leading to body sizes below the reproduction threshold. As a result, one-year-old amphipods have to postpone their reproduction until the following year^35,67^ thus increasing the risk of predation. The Bothnian Sea population crashed in the early 2000s when phytoplankton biomass was low for several consecutive years^68^ resulting in deteriorated condition status for higher trophic levels^69^. In the last decade, primary production has been more stable and the amphipod fecundity has increased in the Bothnian Sea^69^. This increase coincided with an increase in cyanobacteria abundance in this basin^8,70^. Our findings demonstrating improved body condition and growth in the amphipods fed with the cyanobacteria-diatom mixture provide a plausible mechanistic explanation for the amphipod population dynamics during the last decades (Fig. S4, Supplementary Information).

## Conclusions

Our results indicate that the benthic key-species *Monoporeia affinis* has a considerable capacity for coping with cyanobacteria northward expansion, a predicted consequence of the ongoing climate change which is already evident in the Baltic Sea. Both amphipod populations showed an increase in body size with no adverse effects on the body condition index (C:N ratio) when offered a mixed diet (diatoms and cyanobacteria), suggesting a generalist feeding. Contrary to the expectations, we observed a plastic feeding behavior in the Bothnian Sea amphipods, consuming both diatoms and cyanobacteria, with a potential for their trophic niche expansion. However, there were indications of neurotoxicity and cyanotoxin accumulation from a cyanobacteria-rich diet, which warrants further studies on the effects of cyanobacterial bloom frequency and magnitude on the primary consumers, deposit-feeders, and their predators. Our results provide insights to physiological adaptation to the increasing cyanobacteria in future climates in both limnic and marine ecosystems. Also, they might be applicable to other systems and consumers where diet adaptations are relevant. Genetic analyses combined with our experimental approach would provide understanding of the evolutionary mechanisms involved.

## Supplementary Information

Supplementary Information.

## Data Availability

Data from the Dryad Digital Repository (https://doi.org/10.5061/dryad.xwdbrv1fc).
