# Supplementary material for: High capacity for a dietary specialist consumer population to cope with increasing cyanobacterial blooms
Source: Sci Rep. 2022 Dec 22;12:22169. doi: 10.1038/s41598-022-26611-2 (PMC9780316; doi:10.1038/s41598-022-26611-2)
Supplement: Supplementary file 1 — Supplementary Information. [file 41598_2022_26611_MOESM1_ESM.docx]

**Supplementary Information**

**High capacity for a dietary specialist consumer population to cope with increasing cyanobacterial blooms**

Ledesma, M.^1^, Gorokhova, E.^2^, Garbaras, A.^3^, Röjning, L.^1^, Brena, B.^4^, Karlson, A.M.L.^1,5,6^

*¹ Dept. Ecology, Environment and Plant Science (DEEP), Stockholm University, Svante Arrhenius St. 20, Stockholm, Sweden.*

*² Stockholm University, Dept. Environmental Science, Stockholm University, Svante Arrhenius St. 8, Stockholm, Sweden.*

*³ Centre for Physical Science and Technology, Mass Spectrometry Laboratory, Savanorių ave. 231, Vilnius, Lithuania.*

*^4^ Department of Biosciences, University of the Republic, Gral. Flores 2124, Montevideo, Uruguay.*

*^5^ Stockholm University Baltic Sea Centre, Stockholm University, Svante Arrhenius St. 20, Stockholm, Sweden.*

*^6^ Bolin Centre for Climate Research, Stockholm University,* Svante Arrhenius St. 8, *Stockholm, Sweden.*

**Content (in order of appearance in the manuscript)**

**Figure S1**: Survival in the experimental treatments.

**Table S1**: Mean values and differences between treatments for the isotope data.

**Table S2**: Two-way ANOVA results: diet and basin effect on δ^13^C in amphipods.

**Table S3**: Mean values and differences between treatments for growth and body condition data.

**Table S4**: Two-way ANOVA results diet and basin effect on the various growth and condition proxies used in the experiment.

**Table S5:** Mean values and differences between treatments for Acetylcholinesterase activity **Table S6**: Two-way ANOVA output from Acetylcholinesterase activity.

**Table S7**: Two-way ANOVA output from the Nodularin concentration in amphipods.

**Figure S2**: Nodularin concentrations in amphipods

**Table S8**: Layman metrics for the different treatments and populations.

**Figure S3**: Water chemistry in the experimental treatments.

**Figure S4**: Time series for *Monoporeia affinis* fecundity and cyanobacteria biovolume.

**
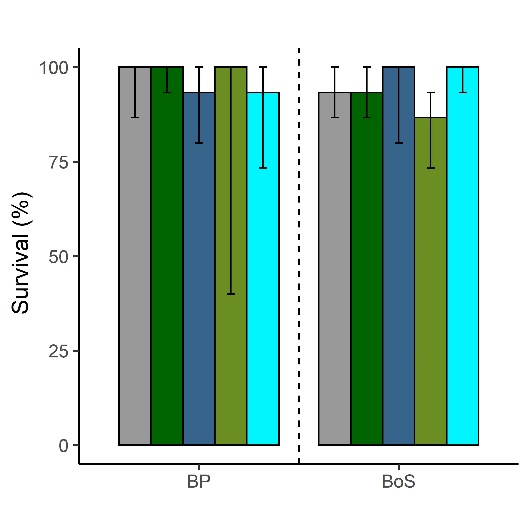
**

**Figure S1**. Survival (%) for each population in the different treatments at the end of the experiment. Colour represents each treatment: Control (grey), HD (green), HDLC (blue), LD (olive green), LDHC (turquoise). Values are median values with maximum and minimum values shown as error bars.

**Table S1**: Mean values for the amphipod stable isotope values (δ^15^N and δ^13^C), absolute difference in per mille between the treatments and percent change relative to the respective controls (S) and between mixed and diatom treatments.

| Endpoint | Basin | Treatment | Mean  (‰) | Difference  between treatments and control (‰) | Difference relative to diatom (‰) |
| --- | --- | --- | --- | --- | --- |
| **δ^15^N** | **BoS** | Initial | 7.01 |  |  |
|  |  | Control | 8.03 | 1.01 |  |
|  |  | HD | 8.73 | 0.71 |  |
|  |  | HDLC | 8.35 | 0.32 | -0.39 |
|  |  | LD | 8.42 | 0.39 |  |
|  |  | LDHC | 6.67 | -1.36 | -1.75 |
|  | **BP** | Initial | 6.83 |  |  |
|  |  | Control | 7.49 | 0.65 |  |
|  |  | HD | 8.70 | 1.21 |  |
|  |  | HDLC | 7.21 | -0.46 | -1.67 |
|  |  | LD | 7.41 | -0.08 |  |
|  |  | LDHC | 6.21 | -1.24 | -1.16 |
| **δ^13^C** | **BoS** | Initial | -23-97 |  |  |
|  |  | Control | -23.74 | 0.23 |  |
|  |  | HD | -23.45 | 0.30 |  |
|  |  | HDLC | -23.50 | 0.24 | -0.05 |
|  |  | LD | -23.42 | 0.33 |  |
|  |  | LDHC | -23.55 | 0.19 | -0.14 |
|  | **BP** | Initial | -23.89 |  |  |
|  |  | Control | -22.69 | 1.20 |  |
|  |  | HD | -22.82 | -0.13 |  |
|  |  | HDLC | -23.00 | -0.31 | -0.19 |
|  |  | LD | -22.67 | 0.01 |  |
|  |  | LDHC | -23.06 | -0.37 | -0.38 |

**Table S2***:* Two-way ANOVA model testing effect of diatoms and cyanobacteria in the diet (a: *diatom*; b and c: *cyano*) and *basin* (BoS vs. BP) as main factors and their interactions (*basin* × *diatom* or *basin* × *cyano*) on δ^13^C values in amphipods. See Table and Fig. 2 for direction of effects.

| Variables | SS | df | MS | F | p-value |  |
| --- | --- | --- | --- | --- | --- | --- |
| **δ^13^C** |  |  |  |  |  |  |
| **a) Diatom input (3 levels: S, LD, and HD)** | | | | | | |
| *diatom* | 0.299 | 2 | 0.15 | 0.909 | 0.412 |  |
| *basin* | 6.599 | 1 | 6.599 | 40.131 | **0.000** | BoS > BP |
| *basins* × *diatom* | 0.319 | 2 | 0.159 | 0.97 | 0.389 |  |
| Residuals | 5.756 | 35 | 0.164 |  |  |  |
| **b) Low diatoms, high proportion of cyanobacteria (LDHC *vs*. LD)** | | | | | | |
| *cyano* | 0.468 | 1 | 0.468 | 3.161 | *0.088* |  |
| *basin* | 2.666 | 1 | 2.661 | 18.006 | **0.000** | BoS > BP |
| *basins* × *cyano* | 0.108 | 1 | 0.108 | 0.73 | 0.401 |  |
| Residuals | 3.553 | 24 | 0.148 |  |  |  |
| **c) High diatoms, low proportion of cyanobacteria (HDLC *vs.* HD)** | | | | | | |
| *cyano* | 0.10 | 1 | 1.008 | 0.935 | 0.343 |  |
| *basin* | 2.240 | 1 | 2.240 | 20.783 | **0.000** | BoS > BP |
| *basins* × *cyano* | 0.03 | 1 | 0.031 | 0.293 | 0.593 |  |
| Residuals | 2.587 | 24 | 0.107 |  |  |  |

**Table S3**: Mean values for body condition data (Body mass and C:N ratio), differences between the treatments and % change relative to the respective population control treatment (S) and between mixed and diatom treatments.

| Endpoint | Basin | Treatment | Mean | Percent change relative to control (%) | Percent change between mixed and diatom treatment (%) |
| --- | --- | --- | --- | --- | --- |
| Body mass | BP | $\mathrm{Initial}$ | 0.76 |  |  |
|  |  | Control | 0.79 | 3 |  |
|  |  | HD | 0.80 | 1 |  |
|  |  | $\mathrm{HDLC}$ | 0.86 | 8 | +7 |
|  |  | $\mathrm{LD}$ | 0.81 | 2 |  |
|  |  | $\mathrm{LDHC}$ | 1.00 | 26 | +23 |
|  | BoS | $\mathrm{Initial}$ | 0.89 |  |  |
|  |  | Control | 0.94 | 4 |  |
|  |  | HD | 0.97 | 3 |  |
|  |  | $\mathrm{HDLC}$ | 1.06 | 13 | +10 |
|  |  | $\mathrm{LD}$ | 0.94 | 0 |  |
|  |  | $\mathrm{LDHC}$ | 1.06 | 12 | +13 |
| C:N ratio | BP | $\mathrm{Initial}$ | 7.65 |  |  |
|  |  | Control | 5.506 | -28 |  |
|  |  | HD | 5.54 | 1 |  |
|  |  | $\mathrm{HDLC}$ | 6.16 | 12 | +11 |
|  |  | $\mathrm{LD}$ | 5.51 | 0 |  |
|  |  | $\mathrm{LDHC}$ | 6.33 | 15 | +15 |
|  | BoS | $\mathrm{Initial}$ | 7.06 |  |  |
|  |  | Control | 6.87 | -3 |  |
|  |  | HD | 6.43 | -6 |  |
|  |  | $\mathrm{HDLC}$ | 6.85 | 0 | +7 |
|  |  | $\mathrm{LD}$ | 6.56 | -5 |  |
|  |  | $\mathrm{LDHC}$ | 6.85 | 0 | +5 |

**Table S4***:* Two-way ANOVA model testing effect of diatoms and cyanobacteria in the diet (a: *diatom*; b and c: *cyano*) and *basin* (BoS vs. BP) as main factors and their interactions (*basin* × *diatom* or *basin* × *cyano*) on growth and C:N ratio in amphipods. See Table and Fig. 3 for direction of effects.

| Variables | SS | df | MS | F | p-value |  |
| --- | --- | --- | --- | --- | --- | --- |
| **Growth** | | | | | | |
| **a) Diatom input (3 levels: S, LD, and HD)** | | | | | | |
| *diatom* | 0.002 | 2 | 0.001 | 0.040 | 0.961 |  |
| *basin* | 0.000 | 1 | 0.000 | 0.015 | 0.902 |  |
| *basins* × *diatom* | 0.002 | 2 | 0.001 | 0.050 | 0.951 |  |
| Residuals | 0.818 | 35 | 0.023 |  |  |  |
| **b) Low diatoms, high proportion of cyanobacteria (LDHC *vs*. LD)** | | | | | | |
| *cyano* | 0.161 | 1 | 0.161 | 7.551 | **0.011** | LDHC >LD |
| *basin* | 0.015 | 1 | 0.015 | 0.699 | 0.412 |  |
| *basins* × *cyano* | 0.007 | 1 | 0.007 | 0.348 | 0.561 |  |
| Residuals | 0.512 | 24 | 0.021 |  |  |  |
| **c) Low proportion of cyanobacteria (HDLC vs HD)** | | | | | | |
| *cyano* | 0.039 | 1 | 0.039 | 2.629 | 0.118 |  |
| *basin* | 0.013 | 1 | 0.013 | 0.876 | 0.359 |  |
| *basins* × *cyano* | 0.003 | 1 | 0.003 | 0.198 | 0.660 |  |
| Residuals | 0.359 | 24 | 0.015 |  |  |  |
| **C:N ratio** | | | | | | |
| **a) Diatom input (3 levels: S, LD, and HD)** | | | | | | |
| *diatom* | 0.502 | 2 | 0.251 | 0.660 | 0.523 |  |
| *basin* | 12.280 | 1 | 12.280 | 32.340 | **0.000** | BoS > BP |
| *basins* × *diatom* | 0.379 | 2 | 0.189 | 0.499 | 0.612 |  |
| Residuals | 13.291 | 35 | 0.380 |  |  |  |
| **b) Low diatoms, high proportion of cyanobacteria (LDHC *vs*. LD)** | | | | | | |
| *cyano* | 2.201 | 1 | 2.201 | 9.261 | **0.006** | LDHC > LD |
| *basin* | 4.329 | 1 | 4.329 | 18.217 | **0.000** | BoS > BP |
| *basins* × *cyano* | 0.481 | 1 | 0.481 | 2.024 | 0.168 |  |
| Residuals | 5.703 | 24 | 0.238 |  |  |  |
| **c) Low proportion of cyanobacteria (HDLC vs HD)** | | | | | |  |
| *cyano* | 1.908 | 1 | 1.908 | 8.166 | **0.009** | HDLC > DH |
| *basin* | 4.392 | 1 | 4.392 | 18.795 | **0.000** | BoS > BP |
| *basins* × *cyano* | 0.075 | 1 | 0.075 | 0.321 | 0.576 |  |
| Residuals | 5.609 | 24 | 0.234 |  |  |  |

**Table S5**: Mean values of AChE activity (lower mean values indicate inhibition and a neurotoxic effect) for each population and treatment and difference (increase or inhibition) in percentage relative to the control and between mixed and diatom treatments

| Endpoint | Population | Treatment | Mean | Change relative to control (%) | Change between mixed and diatom treatment (%) |
| --- | --- | --- | --- | --- | --- |
| AChE activity | BP | Control | 12.6 |  |  |
|  |  | HD | 14.4 | 14 |  |
|  |  | $\mathrm{HDLC}$ | 10.4 | -17 | -28 |
|  |  | $\mathrm{LD}$ | 14.3 | 13 |  |
|  |  | $\mathrm{LDHC}$ | 13.7 | 9 | -4 |
|  | BoS | Control | 11.4 |  |  |
|  |  | HD | 10.9 | -4 |  |
|  |  | $\mathrm{HDLC}$ | 9.87 | -13 | -9 |
|  |  | $\mathrm{LD}$ | 12 | 5 |  |
|  |  | $\mathrm{LDHC}$ | 9.45 | -17 | -21 |

**Table S6**. Two-way ANOVA model testing effect of diatoms and cyanobacteria in the diet (a: *diatom*; b and c: *cyano*) and *basin* (BoS vs. BP) as main factors and their interactions (*basin* × *diatom* or *basin* × *cyano*) on the acetylcholinesterase (AChE) activity in amphipods. See Table and Fig. 4 for direction of effects.

| Variables | SS | df | MS | F | p-value |  |
| --- | --- | --- | --- | --- | --- | --- |
| **AChE activity** | | | | | |  |
| **a) Diatom input (3 levels: S, LD, and HD)** | | | | | |  |
| *diatom* | 0.061 | 2 | 0.031 | 0.211 | 0.811 |  |
| *basin* | 0.476 | 1 | 0.476 | 3.288 | *0.078* |  |
| *basins* × *diatom* | 0.110 | 2 | 0.055 | 0.379 | 0.687 |  |
| Residuals | 5.214 | 36 | 0.145 |  |  |  |
| **b) High proportion of cyanobacteria (LDHC vs LD)** | | | | | |  |
| *cyano* | 0.192 | 1 | 0.192 | 1.421 | 0.245 |  |
| *basin* | 0.591 | 1 | 0.591 | 4.368 | **0.047** | BP > BoS |
| *basins* × *cyano* | 0.111 | 1 | 0.111 | 0.819 | 0.374 |  |
| Residuals | 3.247 | 24 | 0.135 |  |  |  |
| **c) Low proportion of cyanobacteria (HDLC vs HD)** | | | | | |  |
| *cyano* | 0.139 | 1 | 0.139 | 1.112 | 0.302 |  |
| *basin* | 0.282 | 1 | 0.282 | 2.248 | 0.147 |  |
| *basins* × *cyano* | 0.167 | 1 | 0.167 | 1.331 | 0.260 |  |
| Residuals | 3.011 | 24 | 0.126 |  |  |  |

**Table S7**. Two-way ANOVA testing effect of cyanobacterial addition (LDHC treatment only) relative to control and basin on nodularin concentration (ng/g) in amphipods. See Fig. S2 for direction of effects.

| Variables | SS | df | MS | F | p-value |
| --- | --- | --- | --- | --- | --- |
| *basin* | 30691 | 1 | 30691 | 6.642 | **0.033** |
| *diet* (LDHC vs. control) | 15848 | 1 | 15848 | 3.43 | 0.101 |
| *basin* × *diet* | 386 | 1 | 386 | 0.084 | 0.780 |
| Residuals | 36967 | 8 | 4621 |  |  |


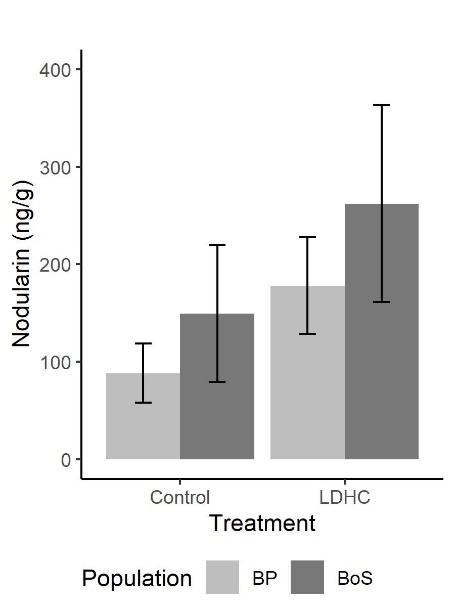


**Figure S2**. Nodularin concentrations in the BP and BoS amphipods from control and LDHC treatments. Values are mean ± SD.

**Table S8**. Layman metrics (NR, CR, TA) shown as absolute values for the initials and in the experimental treatments as % fold increase (+ and – indicating increase and decrease, respectively) in relation to the diatom treatment in each basin.

|  | NRb | | CRb | | TAb | |
| --- | --- | --- | --- | --- | --- | --- |
| Basins | BP | BoS | BP | BoS | BP | BoS |
| Initial | 2.27 | 1.94 | 1.32 | 0.78 | 2.13 | 0.82 |
| Treatment |  |  |  |  |  |  |
| Control | 4.17 | 3.79 | 2.78 | 2.33 | 6.03 | 4.61 |
| HD vs. HDLC | 0.77 | -2.16 | 0.55 | -1.43 | 6.92 | 1.60 |
| LD vs. LDHC | 0.15 | 0.11 | 0.15 | 0.11 | 5.36 | 2.09 |

dN and dC , the range of the δ^15^N values and δ^13^C , respectively; TA, total area of the convex hull.


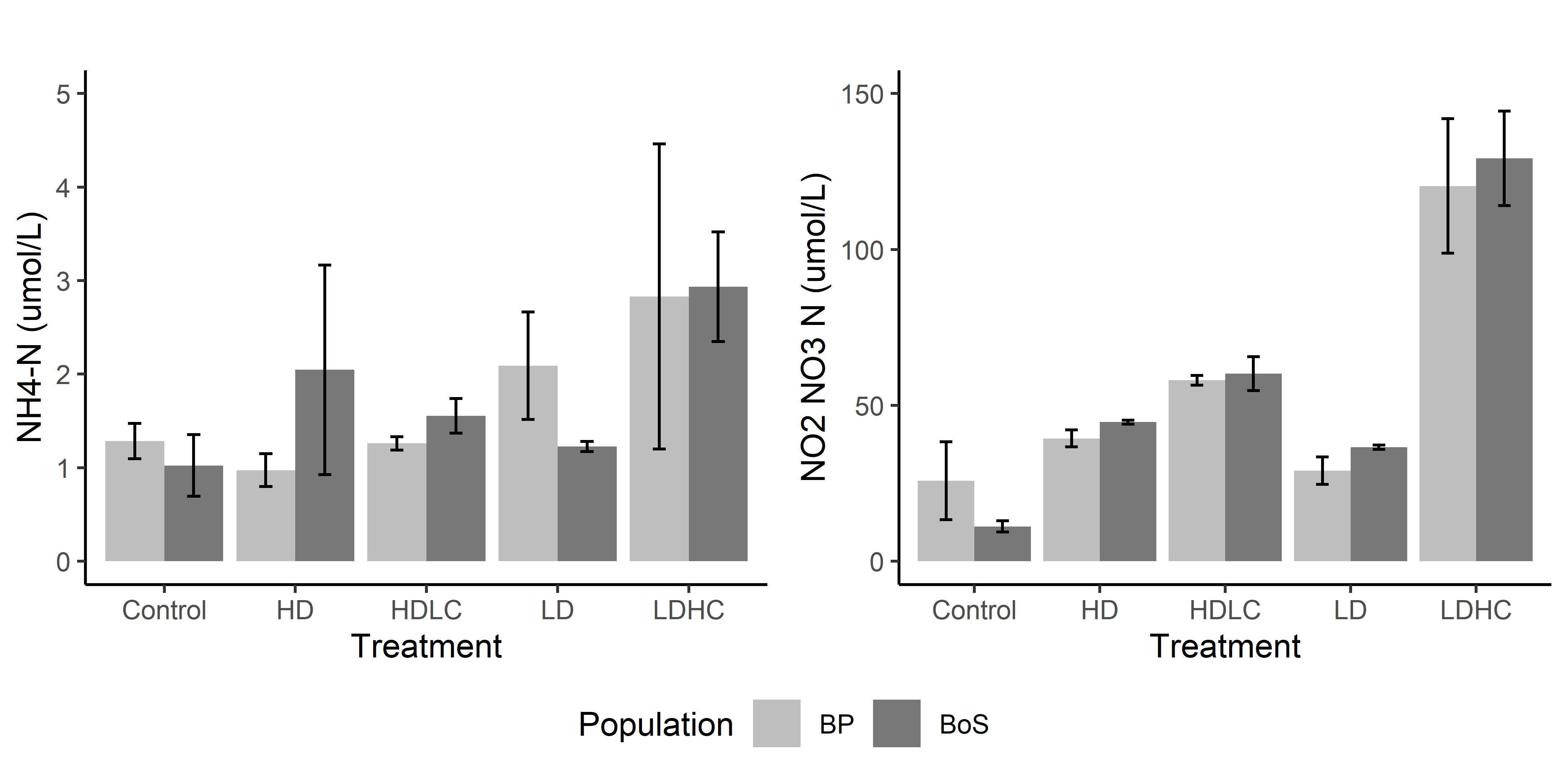


**Figure S3**: Ammonium (NH4-N) and nitrate (NO2 NO3) concentrations for each treatment and population. All treatments have ammonium concentration below the toxic level (96 hr LC_50_ 1000 – 2000 µmol 1 ^-^¹, for *Hyalella azteca*, Ankley et al., 1995). Values are mean ± SD.


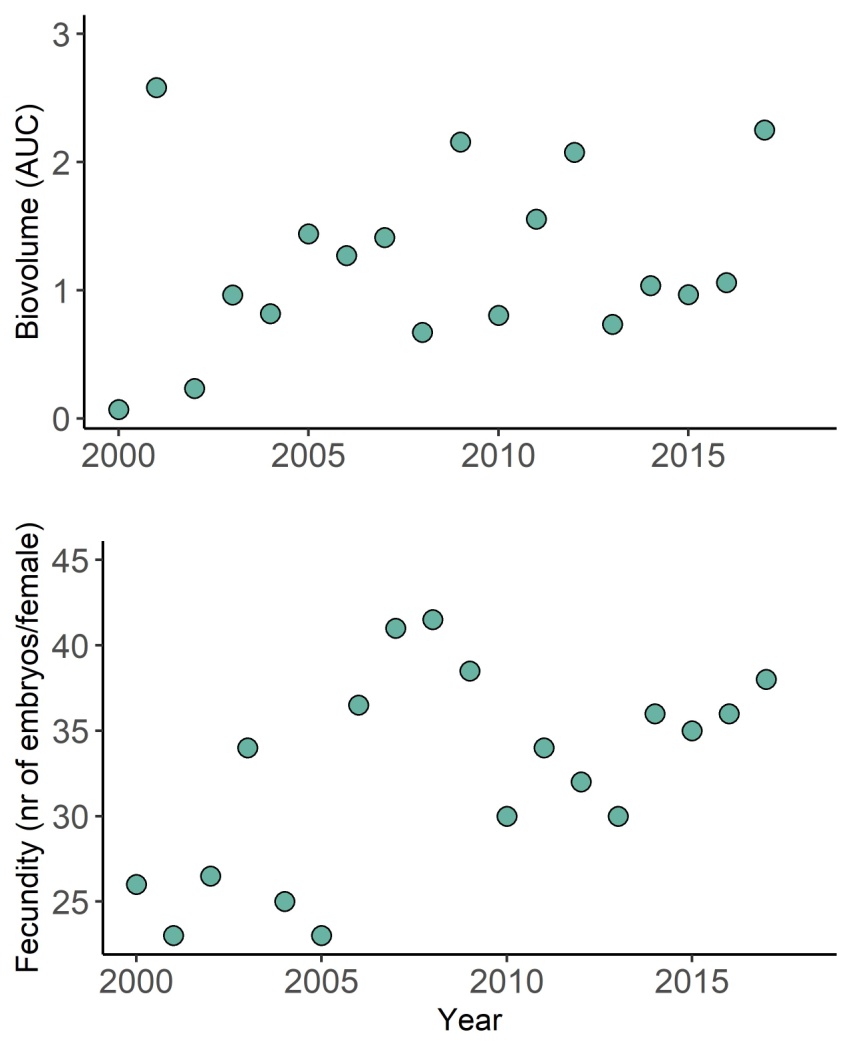


**Figure S4:** Upper panel shows annual total biovolume of the three species of N-fixing cyanobacteria in the Baltic (*Aphanizomenon sp, Dolichospermum spp, Nodularia spumigena*) calculated from three stations from the northern Bothnian Sea (B3: 63º29.98'N;19º49.14'E, B7: 63º31.50'N;19º48.49'E, C3: 62º39.17'N; 18º57.14'E) for the time period 2000-2017 (Olofsson et al. 2020, M. Olofsson kindly provided rawdata). In the lower panel, *Monoporeia affinis* median fecundity from three stations (N25: 63°19'9.98"N;19°48'20.02"E, N26: 63°12'50.00"N;19°38'15.00"E; N27: 63° 5'42.00"N; 19°32'49.99"E) for the same time period as above (data from the national monitoring program on reproductive disorders, data publicly available from www.sgu.se). Monitoring data of the biovolume (mm^3^ L^-1^) is calculated for the period May – September from each year as area under the curve (AUC) using the R package “*bayestestR*” by Makowski et al. (2019). In the biovolume graph, the time line is shifted one year earlier relative to the fecundity (measured in January), to better match the relevant feeding season. Mann-Kendall test showed positive trends for both fecundity (r_τ_= 0.35, p < 0.03) and biovolume (r_τ_= 0.215, p > 0.11).

**References:**

Makowski D, Ben-Shachar M, Lüdecke D (2019). “bayestestR: Describing Effects and their Uncertainty, Existence and Significance within the Bayesian Framework.” *Journal of Open Source Software*, 4(40),1541.doi: 10.21105/joss.01541

Olofsson, M., S. Suikkanen, J. Kobos, N. Wasmund, and B. Karlson. 2020. Basin-specific changes in filamentous cyanobacteria community composition across four decades in the Baltic Sea. Harmful Algae 91: 101685. doi: 10.1016/j.hal.2019.101685
